# Grb7 Protein Stability Modulated by Pin1 in Association with Cell Cycle Progression

**DOI:** 10.1371/journal.pone.0163617

**Published:** 2016-09-22

**Authors:** Yu-Ling Tai, Li-Hsuan Tung, Yu-Chi Lin, Pei-Jung Lu, Pei-Yu Chu, Ming-Yang Wang, Wei-Pang Huang, Ko-Chien Chen, Hsinyu Lee, Tang-Long Shen

**Affiliations:** 1 Department of Plant Pathology and Microbiology, National Taiwan University, Taipei, Taiwan; 2 Institute of Clinical Medicine, Medical College, National Cheng Kung University, Tainan, Taiwan; 3 Department of Genetics, Yale Stem Cell Center, Yale School of Medicine, Connecticut, United States of America; 4 Department of Surgery, National Taiwan University Hospital, Taipei, Taiwan; 5 Institute of Zoology, National Taiwan University, Taipei, Taiwan; 6 Center for Biotechnology, National Taiwan University, Taipei, Taiwan; Indian Institute of Science Education and Research, INDIA

## Abstract

Growth factor receptor bound protein-7 (Grb7) is a multi-domain adaptor protein that is co-opted by numerous tyrosine kinases involved in various cellular signaling and functions. The molecular mechanisms underlying the regulation of Grb7 remain unclear. Here, we revealed a novel negative post-translational regulation of Grb7 by the peptidyl-prolyl *cis/trans* isomerase, Pin1. Our data show that phosphorylation of Grb7 protein on the Ser^194^-Pro motif by c-Jun N-terminal kinase facilitates its binding with the WW domain of Pin1. Subsequently, Grb7 is degraded by the ubiquitin- and proteasome-dependent proteolytic pathway. Indeed, we found that Pin1 exerts its peptidyl-prolyl *cis/trans* isomerase activity in the modulation of Grb7 protein stability in regulation of cell cycle progression at the G2-M phase. This study illustrates a novel regulatory mechanism in modulating Grb7-mediated signaling, which may take part in pathophysiological consequences.

## Introduction

Growth factor receptor bound protein 7 (Grb7) is a member of the Grb7 adaptor protein family that includes Grb10 and Grb14 proteins. The entire Grb7 family proteins are composed of five major protein-binding modules, including an N-terminal proline-rich region, a putative RA (Ras-associating) domain, a central PH (pleckstrin homology) domain, a BPS motif (between PH and SH2 domains), and a C-terminal SH2 domain [1–3]. Although devoid of any enzymatic activity, these protein-binding modules enable Grb7 through simultaneous interactions with growth and/or adhesion receptors as well as intracellular proteins. Such interaction further facilitates the formation of signaling complexes involved in multiple signal transduction cascades that set forth to regulate diverse cellular functions [1, 2]. While, the physiological roles of these interactions are defined under certain pathological states, the detailed molecular mechanism of Grb7 regulation has not yet been elucidated.

Several studies have suggested that the tyrosine phosphorylation state of Grb7 is crucial for its regulation and functionality. Various stimuli, such as epidermal growth factor [4], ephrin type-B receptor 1 [5], extracellular matrix [6], and focal adhesion kinase [7, 8] were shown to exert influences on the tyrosine phosphorylation state of Grb7, and can further modulate cell migration, cell proliferation as well as tumorigenicity [4, 8]. Conversely, serine/threonine phosphorylation is thought to be constitutive but less understood in Grb7 [2]. Nevertheless, some studies have indicated that the phosphorylation of serine/threonine residues preceding proline (i.e., phospho-Ser/Thr–Pro) is a critical for modulating protein conformation, stability and its cellular functions, like cell proliferation and cell transformation [9–12]. In fact, there are nine serine/threonine residues preceding proline within Grb7 protein. Nevertheless, whether phosphorylation of serine/threonine residues preceding proline will affect protein stability and functionality of Grb7 is unclear.

The peptidyl-prolyl *cis/trans* isomerase, Pin1, is an essential regulator for multiple post-translational modifications by catalyzing the conversion of phospho-Ser/Thr–Pro motifs between two distinct *cis* and *trans* isomers of a protein [13]. Pin1 contains two functional domains, an N-terminal WW domain that binds certain phospho-Ser/Thr–Pro motifs and a C-terminal PPIase domain with specific catalytic activity for *cis/trans* isomerization of peptidyl-prolyl peptide bonds [14]. Pin1 isomerizes specific phosphorylated Ser/Thr–Pro motifs to modulate protein functions, such as protein stability [12, 15], protein binding ability [16], protein localization [17], phosphorylation state [18], and the transcriptional activity of transcription factors [19]. As a result, Pin1 serves as an important mediator in regulating physiological processes and pathological conditions, such as the cell cycle, cell proliferation, cell apoptosis, Alzheimer’s disease and cancer [12, 15, 17, 20–22]. Taken together, these studies indicate that the phosphorylation-specific isomerase Pin1 is a critical turning point in post-translational modifications and functional alterations.

In the present study, we first identified a serine phosphorylation site preceding a proline residue, Ser194, on Grb7 protein. This phosphorylation was catalyzed by JNK, which enables interaction with Pin1 via its WW domain. Then, the interaction between Grb7 and Pin1 then subjects Grb7 ubiquitination and subsequent degradation through proteasome-mediated proteolysis in a Pin1 isomerase activity-dependent manner. Consequently, we revealed Pin1 involved in Grb7-mediated cell cycle progression.

## Materials and Methods

### Reagents and antibodies

Glutathione-agarose beads, protein A-sepharose 4B beads, human plasma fibronectin, poly-L-lysine, EGF, G-418 disulfate salt, 5’-bromo-2-deoxyuridine (BrdU), puromycin, cycloheximide, LY294002, and SB431542 were purchased from Sigma-Aldrich (St Louis, MO). His-Tag binding agarose was purchased from Bioman Scientific (Taipei, Taiwan). SB203580, PD98059, JNK inhibitor II and MG-132 were obtained from Calbiochem (Darmstadt, Germany). CGP74514A, SB-415286, and TDZD-8 were gifts form Dr. Pei-Jung Lu (Institute of Clinical Medicine, National Cheng Kung University, Taiwan). Fetal bovine serum (FBS), Dulbecco’s modified Eagle’s medium (DMEM), RPMI, DMEM/F-12 (1:1), and Opti-MEM media were purchased from Invitrogen (Carlsbad, CA). The mouse monoclonal anti-Cyclin D1 (5D4, ADI-KAM-CC200-E) antibody was purchased from Enzo Life Science (Farmingdale, NY). The mouse monoclonal anti-Akt-1 (B-1, sc-5298), anti-HA (F-7, sc-7392), and anti-GFP (B-2, sc-9996) antibodies were obtained from Santa Cruz Biotechnology (Santa Cruz, CA). The rabbit polyclonal anti-Grb7 (N-20, sc-607), anti-Grb7 (C-20, sc-606), anti-JNK (FL, sc-571), anti-ERK 1 (C-16, sc-93), anti-p38α (N-20, sc-728), anti-HA-probe (Y-11, sc-805), and anti-Pin1 (H-123, sc-15340) antibodies were obtained from Santa Cruz Biotechnology (Santa Cruz, CA). The rabbit polyclonal anti-phospho-cdc2 (Thr161, 9114S), the rabbit polyclonal anti-phospho-Erk1/2 (Thr202/Tyr204, 9101), the mouse monoclonal anti-phospho-SAPK/JNK (Thr183/Tyr185, 9255), the rabbit monoclonal anti-GSK-3β (27C10, 9315), and the rabbit monoclonal anti-phospho-GSK-3α/β (Ser21/9, 9331) antibodies were purchased from Cell Signaling (Danvers, MA). The rabbit monoclonal anti-phospho-p38α (Thr180/Tyr182, 05–1059), the mouse monoclonal anti-phospho-Ser/Thr motif (MPM2, 05–368) and anti-actin (C4, MAB1501) were obtained from Millipore (Billerica, MA). The mouse monoclonal anti-BrdU (BU-33, B2531), anti-Flag (M2, F3165), and anti-GST (GST-2, G1160) antibodies were purchased from Sigma-Aldrich (St Louis, MO). The secondary horseradish peroxidase-conjugated goat anti-rabbit antibodies and goat anti-mouse antibodies were obtained from Jackson ImmunoResearch Laboratories (West Grove, PA).

### Yeast two-hybrid

The human brain Matchmaker cDNA library (cultured in *Saccharomyces cerevisiae* strain Y187), pGBTK7 (GAL4 DNA-binding domain vector), pGADT7 (GAL4 activation domain vector), and *Saccharomyces cerevisiae* strain AH109 were obtained from Clontech Laboratories (Mountain View, CA). The full length of Grb7 was used as a bait protein. A total of 1.4 million independent clones from the human brain cDNA library constructed in yeast strain Y187 were screened with yeast strain AH109 transformed with pGBTK7-Grb7. The combination of 1 ml of library strain Y187 and 5 ml of bait strain AH109 pGBTK7-Grb7 was cultured in a sterile 2-liter Erlenmeyer flask with 45 ml of 2 × YPDA (containing kanamycin) at 30°C for 20–24 hr. The mating culture was washed with 0.5 × YPDA (containing kanamycin) and resuspended in 0.5 × YPDA (containing kanamycin). Serial dilutions of yeast culture were spread on SD/-Leu, SD/-Trp, and SD/-Leu/-Trp plates and incubated at 30°C for 3–5 days to determine the mating efficiency. We streaked the remaining culture first on SD/-His/-Leu/-Trp plates at 30°C for one week, and then, we transferred all the colonies to SD/-Ade/-His/-Leu/-Trp plates by replica plating and incubated the plates at 30°C for one week. The library proteins that bind with pGBTK7 were confirmed by retreating on SD/-Ade/His/-Leu/-Trp agar plates, as well as by X-gal assay. All the positive clones were transformed into *Escherichia coli* strain DH5α to obtain the library plasmids, which were then sequenced. After sequencing, the sequences were subjected to a BLAST search in the National Center for Biotechnology Information database to find all the matching genes.

### Cell culture

A431 human epithelial carcinoma and 293T human epithelial kidney cell lines were gifts from Dr. Jun-Lin Guan (Univeristy of Cincinnati, Cincinnati, OH, USA) and maintained in Dulbecco's modified Eagle's medium (DMEM) supplemented with 10% fetal bovine serum (Invitrogen, Carlsbad, CA). Pin1 wild-type (Pin1^+/+^) and knockout (Pin1^-/-^) mouse embryonic fibroblasts were gifts form Dr. Pei-Jung Lu (Institute of Clinical Medicine, National Cheng Kung University, Tainan, Taiwan) and maintained in Dulbecco's modified Eagle's medium (DMEM) supplemented with 10% fetal bovine serum (Invitrogen, Carlsbad, CA). All cells were incubated in a 37°C humidified 5% CO2 incubator. Cells were transfected with mammalian expression plasmids, as indicated, using Lipofectamine 2000^TM^ transfection reagent (Invitrogen, Carlsbad, CA) according to the manufacturer’s instructions. Experiments were conducted 24–48 hr after transfection. Transfected cells were selected by G418 (Sigma-Aldrich, St Louis, MO).

### Construction of expression vectors

The expression plasmids encoding Grb7 or Pin1, including pKH3-Grb7, pEGFP-C3-Grb7, pGEX-2T-Grb7, and pDNR-Dual-PIN1 (which was purchased from the DF/HCC DNA Resource Core, Harvard Medical School), were used as a template; individual point mutation was generated by site-directed mutagenesis, as described previously [8], using the following primer sets (underlining represents the mutated amino acid sequences): 5′-GCGAATTCATGGAGCTGGATC-3′ (sense) and 5′-GGACAAACCACAACTAGAATGCAG-3′ (anti-sense) for Grb7; 5′- TTCAAGAGCGCCCCACACTCC-3′ (sense) and 5′-GGAGTGTGG GGCGCTCTTGAA-3′ (antisense) for Grb7 S194A; 5′-TGTTTGGGCGCCCCACCCTTG-3′ (sense) and 5′- CAAGGGTGGGGCGCCCA AACA-3′ (antisense) for Grb7 S361A; 5′- CCGAATTCGGGGCGGCTGGCATC-3′ (sense) and 5′- CAGAATTCTTAGGGGCGGCTGGCATC-3′ (anti-sense) for the Grb7-N domain; 5′-ATGGATCCATGGCGGACGAGGAG-3′ (sense) and 5′-GAGAATTCTCACTCAGTGCGGAGG-3′ (antisense) for Pin1; 5′- TAACGCCAGCCAGGCGGAGCG-3′ (sense) and 5′- CGCTCCGCCTGGCTGGCGTTA-3′ (antisense) for Pin1 W34A; 5′-ACCTGCTGGTGGCGCACAG-3′ (sense) and 5′-GACTGGCTGTGCGCCACCA-3′ (antisense) for Pin1 K63A; 5′-TAGCATGACTGGTGGACAGC- 3′ (sense) and 5′-CAAGAA TTCTCAACCACTGCTGCTGTTGC-3′ (antisense) for the Pin1-WW domain; 5′-CAAGGATCCAGCAGTGGTGGCAAAAACG-3′ (sense) and 5′-TGTCGACGGA GCTCGAAT-3′ (anti-sense) for the Pin1-PPIase domain. The final PCR products from pKH3-Grb7 were digested with EcoRI and BamHI and then inserted into the pKH3 vector. The final PCR products from pKH3-Grb7 were digested with EcoRI and then inserted into the pGBTK7 vector. The final PCR products from pDNR-Dual-Pin1 were digested with EcoRI and BamHI and then inserted into the pKH3 or pGEX-2T vector. The pKH3-Grb7 ΔN domain was subcloned from pGEX2T-Grb7 by EcoRI into the pKH3 vector. The pGEX-2T-Pin1 W34A was subcloned from pKH3-Pin1 W34A by EcoRI and BamHI. pFlag-Ubiquitin was a gift form Dr. Tsai-Kun Li (Graduate Institute of Microbiology, National Taiwan University, Taipei, Taiwan). pCR-FLAG-MKK7 was a gift from Dr. Jaw-Ji Yang (School of Dentistry, Chung Shan Medical University, Taichung, Taiwan).

### RNA extraction and reverse-transcription PCR

RNA was extracted from cells using MaestroZol^TM^ RNA extraction reagent (Maestrogen, Nevada, USA). The cDNA was synthesized from RNA using M-MLV reverse transcriptase according to the manufacturer’s recommendations (Invitrogen, Carlsbad, CA). The primers were designed as follows: 5′-GAATTCGCCCCCATGTAGTAAAG-3′ (sense) and 5′-GAATTCGTTTTTCCGGAAGACGAA-3′ (antisense) for Grb7; 5′-GGAGCTGATCAACGGCTACA-3′ (sense) and 5′-TCAGTGCGGAGGATGATGT-3′ (antisense) for Pin1. The housekeeping gene GAPDH was used as a reference for normalization. Quantitation of PCR products was estimated by SYBR Green reagent (5X SYBR Green qPCR Master Mix; Fermentas, Waltham, MA) using the ABI StepOnePlus Real-Time PCR system, and the data were analyzed with ABI StepOnePlus Real-Time PCR software according to the manufacturer’s instructions. qPCRs were performed in triplicate, and copy number alterations were scored as validated if the 2^ΔCt^ was ≥ 2 (gain) or ≤ 0.5 (loss) with CV ≤ 15% of 2^ΔCt^. The Grb7 and Pin1 gene expression levels were normalized by GAPDH, which was used as a reverse transcription control.

### Lentiviral production and infection

Lentiviruses encoding GRB7, PIN1, or LUCIFERASE small-hairpin RNA (shRNA) were obtained from the TRC lentiviral shRNA library in the National RNAi Core Facility of Academia, Taiwan. The targeting sequencings of various shRNAs were as follows: GRB7 shRNA (clone ID: TRCN0000061387) 5′-CCAGGGCTTTGTCCTCTCTTT-3′; PIN1 shRNA (clone ID: TRCN0000010577) 5′-GCCATTTGAAGACGCCTCGTT-3′; PIN1 shRNA (clone ID: TRCN0000001034) 5′-CCAGAAGATCAAGTCGGGAGA-3′; MAPK8 shRNA (clone ID: TRCN0000196850) 5’-GTGTCTTCAATGTCAACAGAT-3’. Production and infection of lentiviruses were processed according to the guidelines of the National RNAi Core Facility of Academia Sinica (Taipei, Taiwan).

### Co-immunoprecipitation and Western blot analysis

Cells were washed twice with ice-cold phosphate-buffered saline (PBS) and then lysed with 1% Nonidet P-40 lysis buffer (20 mM Tris, pH 8.0, 137 mM NaCl, 1% Nonidet P-40, 10% glycerol, 1 mM Na_3_VO_4_, 1 mM phenylmethylsulfonyl fluoride, 10 mg/ml aprotinin, and 20 mg/ml leupeptin) on ice for at least 20 min. Next, lysates were collected and clarified by centrifugation at maximum speed for 30 min at 4°C. Immunoprecipitations were carried out by incubating cell lysates with antibodies, as indicated, for 4 hr at 4°C, followed by incubation for an additional 3 hr with protein A-sepharose 4B beads. Then, after washing three times with lysis buffer, immune complexes were resolved using SDS-PAGE. Finally, Western blotting was performed using horseradish peroxidase-conjugated IgG as a secondary antibody and the Western Lightning-ECL system (PerkinElmer Inc., Waltham, MA) for detection. All experiments were conducted at least three independent times.

### Recombinant protein expression and purification

As previously described [23, 24], GST fusion construct pGEX-2T-Pin1, its point mutation, or its truncated mutants were transformed into Escherichia coli strain BL21 and grown at 37°C until an optical density at 600 nm of 0.6 was reached. Then, the bacteria were induced with 0.5 mM isopropyl-β-thiogalactopyranoside (IPTG) at 37°C for additional 3–4 hr; subsequently, cells were pelleted and resuspended with ice-cold PBS following sonication using a Misonix sonicator 3000. Then, a final addition of 1% triton X-100 was performed to the homogenate and incubated on ice for 1 h. The lysates were clarified by centrifugation and immobilized on GST-agarose beads (Sigma-Aldrich, St Louis, MO) at 4°C overnight. Finally, the beads with recombinant GST fusion proteins were washed with PBS.

### Cell migration assay

Cell migration assays were carried out by a Neuro Probe 48-well chemotaxis Boyden chamber (Cabin John, MD) as described previously [8]. Briefly, 5 × 10^4^ cells were trypsinized and resuspended in DMEM including 0.2% FBS and then added to each upper well. Cells were allowed to migrate toward fibronectin (10 μg/ml) in DMEM as the chemoattractant or DMEM only as a control in the lower wells for 7 hr in a 37°C humidified 5% CO_2_ incubator. At the end of the experiments, cells on the upper side of the polycarbonate membrane were removed, and the bottom-side cells were fixed in methanol for 10 min and stained with crystal violet (Sigma-Aldrich, St Louis, MO). The migrated cells were counted in four randomly selected fields of each well under a light microscope (Model IX71, Olympus, Japan) using a 20× objective lens.

### BrdU incorporation assay

Sixteen hours after transfection, cells were first serum starved for 24 hr in DMEM with 0.2% FBS. Cells were then washed with DMEM and incubated for 20 hr in DMEM with 10% FBS and 100 μM BrdU (Sigma-Aldrich, St Louis, MO). Thereafter, cells were fixed in 4% paraformaldehyde for 15 min at room temperature, permeabilized with 0.5% Triton X-100, treated with DNase I (New England Biolabs, Inc., UK), and then processed for immunofluorescence staining with anti-BrdU (1:200, Sigma-Aldrich, St Louis, MO) antibody, as described previously [8] with modifications. Finally, cells were then counted in several fields and scored for BrdU-positive staining in each independent experiment.

### Fluorescence-activated cell sorting analysis

Cells were washed with cold PBS and then fixed in 4% paraformaldehyde at 4°C for 1 hr. Thereafter, cells were washed in cold PBS and treated with propidium iodide staining solution (0.1% Triton-X100, 100 μg/ml RNase A and 20 μg/ml propidium iodide) in the dark for 30 min. Cells were analyzed by flow cytometry (BD, Accuri C6, US).

### Statistical analysis

Student’s t-test was used for statistical analyses. The data in this study were represented as mean ± SD of at least three independent experiments. *, *p* < 0.05 was considered significant differences among the experimental groups.

## Results

### The WW domain of Pin1 binds to the pSer/Thr-Pro motif of Grb7

**Fig 1:**
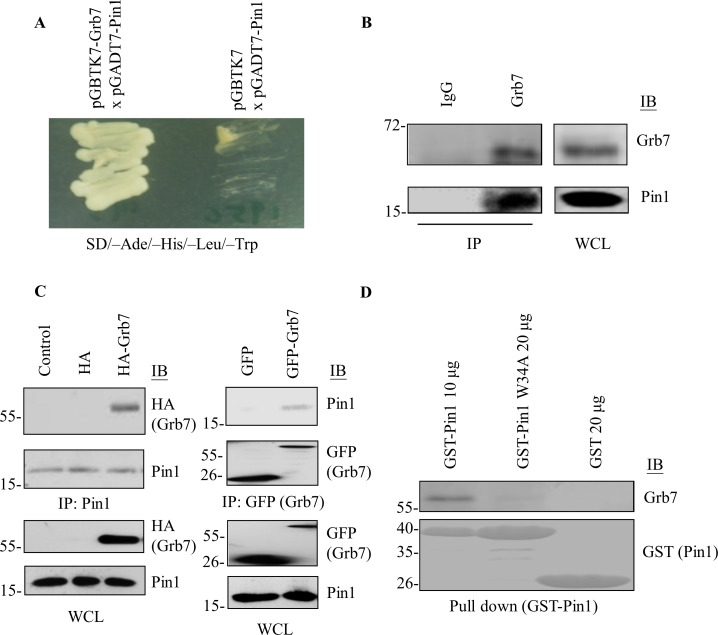
Pin1 interacts with Grb7. (A) pGADT7-Pin1 was co-transformed with pGBTK7 (Mock) or pGBTK7-Grb7 into *Saccharomyces cerevisiae* strain AH109 and was cultured under a SD/-Ade/-His/-Leu/-Trp selective condition. The interaction between Pin1 and Grb7 was detected by a yeast-two hybrid system. (B) Cell lysates from A431 cells were immunoprecipitated by anti-Grb7 antibody, and the co-immunoprecipitated Pin1 was visualized by anti-Pin1 antibody. (C) Cell lysates from HA-Grb7-transfected HEK293 cells were immunoprecipitated by anti-Pin1 antibody, and the co-immunoprecipitated Grb7 was visualized by anti-HA antibody. Reciprocally, cell lysates from GFP-Grb7-transfected A431 cells were immunoprecipitated by anti-GFP antibody against GFP-Grb7; co-immunoprecipitated Pin1 was visualized by anti-Pin1 antibody. (D) A431 cell lysates were separately incubated with different amounts of GST-Pin1 or GST-Pin1-W34A (a phospho-Ser/Thr-Pro motif binding-deficient mutant) recombinant protein (shown on the bottom using Coomassie blue staining). GST-fusion recombinant proteins were pulled down by GST-conjugated beads, followed by Western blotting (IB) with anti-Grb7 antibody. GST proteins were used as the control. Each experiment was repeated at least three independent times. The representative image and blots were shown in A-D.

The Grb7 adaptor protein family exploits its facilitation of protein-protein interactions to participate in intracellular signaling pathways. To better understand the molecular mechanism of Grb7-mediated signal transduction, we attempted to identify Grb7-interacting proteins using a yeast-two hybrid screen, in which Grb7 was used as a bait to screen the human fetal brain cDNA library. Interestingly, the most frequently found Grb7-interacting candidate (11 out of 75 positive candidates) exhibited high homology to the sequence of Pin1 (Fig 1A). This result prompted us to further confirm the interaction between Grb7 and Pin1 in mammalian cells. As shown in Fig 1B, the endogenous Pin1 enabled co-immunoprecipitation by endogenous Grb7 in A431 cells. Furthermore, exogenous HA-tagged Grb7 enabled co-immunoprecipitation with endogenous Pin1 in HEK293 cells. Reciprocally, the exogenous GFP-tagged Grb7 was capable of pulling down endogenous Pin1 (Fig 1C). Moreover, GST-tagged Pin1 recombinant protein (GST-Pin1) purified from *E*. *coli* was capable of efficiently pulling down Grb7 (Fig 1D). Collectively, these results revealed Pin1 as a new binding partner for Grb7.

**Fig 2:**
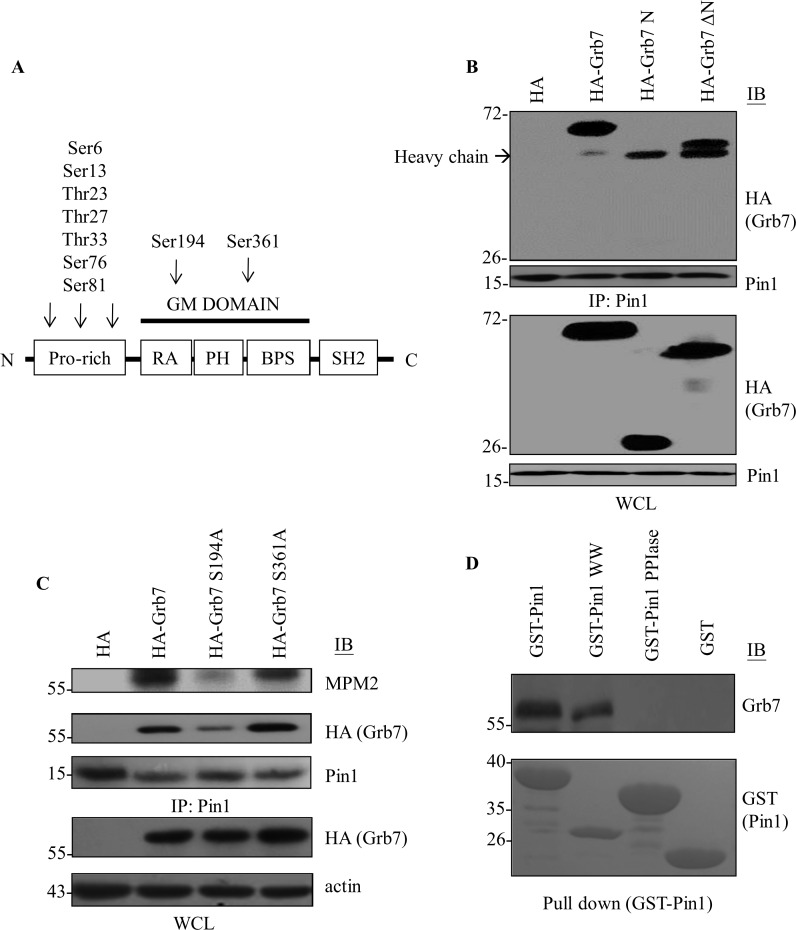
The WW domain of Pin1 interacts with the phospho-Ser/Thr-Pro motif of Grb7. (A) Ser/Thr-Pro motifs on N-terminal domain or GM domain within Grb7 were listed. (B) HEK293 cells were transfected with HA-tagged full-length Grb7, its N-terminal domain (N) or N-terminal deleted domain (ΔN). Cell lysates were immunoprecipitated by anti-Pin1 antibody, and the co-immunoprecipitated Grb7 was visualized by anti-HA antibody. (C) HEK293 cells were transfected with HA-Grb7 or its Ser to Ala point mutation mutants, S194A and S361A. Cell lysates were immunoprecipitated by anti-Pin1 antibody and the co-immunoprecipitated Grb7 and its phosphorylation at Ser/Thr-Pro were visualized by anti-HA and anti-phospho-Ser/Thr-Pro MPM2 antibodies, respectively. (D) A431 cell lysates were separately incubated with different GST-tagged Pin1 recombinant proteins: wild-type or its truncated mutants, WW domain and PPIase domain (shown on the bottom using Coomassie blue staining). GST-fusion recombinant proteins were pulled down by GST-conjugated beads, followed by Western blotting with anti-Grb7 antibody. GST proteins were used as the control. Each experiment was repeated at least three independent times and representative blots were shown in B-D.

We further dissected the binding sites for these two molecules. Pin1 was found to retain interaction with the N-terminal truncated mutant (ΔN) rather than the N-terminus of Grb7 (Fig 2A and 2B). Given the preferential binding to the pSer/Thr-Pro motif for Pin1, there are two potential pSer/Thr-Pro motifs within the GM (Grb and Mig homology) domain, which contains the RA, PH, and BPS domains of Grb7. Then, we employed a site-directed mutagenesis approach to convert serine into alanine, i.e., S194A and S361A, and these constructs were subjected to co-immunoprecipitation assay with Pin1. The result indicated that Pin1 strongly interacts with the phospho-Ser^194^-Pro motif, rather than with the phospho-Ser^361^-Pro motif of Grb7 (Fig 2C). Similarly, the S194A mutant of Grb7 indeed diminished the pSer-Pro expression monitored by MPM2 blotting compared to that of the S361A mutant or wild-type Grb7 (Fig 2C upper panel).

In an *in vitro* pull down assay utilizing the GST-tagged full-length, WW domain (amino acids 1–39), or PPIase domain (amino acids 45–163) of Pin1 recombinant proteins, we found that the WW domain of Pin1 was sufficient to interact with Grb7 (Fig 2D). Such result is consistent with previous results indicating that the WW domain of Pin1 specifically recognizes the pSer/Thr-Pro motif, which within Grb7 is the phospho-Ser^194^-Pro motif. Together, these results illustrate a novel interaction between Pin1 and Grb7 via the WW domain and the phospho-Ser^194^-Pro motif, respectively.

### JNK is responsible for phosphorylating the Ser^194^-Pro motif of Grb7

**Fig 3:**
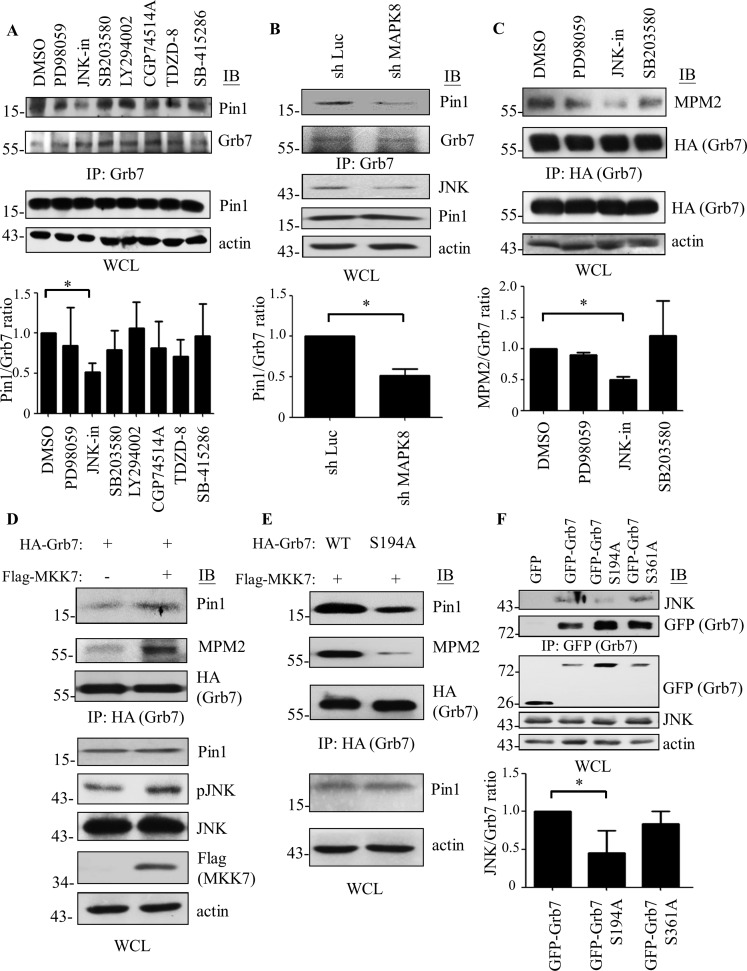
JNK is responsible for phosphorylating the Ser^194^-Pro motif of Grb7. (A) A431 cells were treated with PD98059 (20 μM), JNK-in (25 μM), SB203580 (10 μM), LY294002 (10 nM), CGP74514A (5 μM), TDZD-8 (50 μM), or SB-415286 (20 μM) for 1 hr to detect the effects on the formation of the Pin1/Grb7 complex. Cell lysates were collected and immunoprecipitated by anti-Grb7 antibody, and co-immunoprecipitated Pin1 was visualized by anti-Pin1 antibody. (B) Cell lysates from shLuc- or shMAPK8-infected A431 cells were collected and immunoprecipitated by anti-Grb7 antibody, and co-immunoprecipitated Pin1 was visualized by anti-Pin1 antibody. (C) HA-Grb7-transfected A431 cells were treated with PD98059 (20 μM), JNK-in (25 μM), and SB203580 (10 μM) to detect their effects on the phospho-Ser/Thr-Pro motif of Grb7. Cell lysates were collected and immunoprecipitated by anti-HA antibody, and phosphorylation of the Ser/Thr-Pro motif of Grb7 was visualized by anti-phospho-Ser/Thr-Pro MPM2 antibody. (D) Flag-MKK7 were co-transfected with or without HA-Grb7 into A431 cells. Thirty-six hours after transfection, cell lysates were immunoprecipitated by anti-HA antibody against HA-Grb7, and co-immunoprecipitated Pin1 and phosphorylation of Grb7 on Ser/Thr-Pro motifs were visualized by anti-Pin1 and anti-phospho-Ser/Thr-Pro MPM2 antibodies, respectively. (E) Flag-MKK7 were co-transfected with HA-Grb7 or its Ser to Ala point mutation mutants, S194A, into A431 cells. Thirty-six hours after transfection, cell lysates were immunoprecipitated by anti-HA antibody against HA-Grb7, and co-immunoprecipitated Pin1 and phosphorylation of Grb7 on Ser/Thr-Pro motifs were visualized by anti-Pin1 and anti-phospho-Ser/Thr-Pro MPM2 antibodies, respectively. (F) A431 cells were transfected with GFP-Grb7 or its Ser to Ala point mutation mutants, S194A and S361A. Thirty-six hours after transfection, cell lysates were collected and immunoprecipitated by anti-GFP antibody, and the co-immunoprecipitated JNK was visualized by anti-JNK antibody. The representative blots were shown in A-F. Student’s t-test was used for statistical analyses. Results were represented the mean ± SD of at least three independent experiments. *, *p* < 0.05 was considered significant differences among the experimental groups.

Given that the phospho-Ser^194^-Pro motif of Grb7 is critical for interaction with Pin1, we then asked which kinase is responsible for the phosphorylation of the Ser^194^-Pro motif of Grb7. To resolve this question, several potential Ser/Thr kinase inhibitors, including cyclin-dependent kinase 1 (CGP74514A), GSK-3 (SB-415286), GSK-3β (TDZD-8), AKT (LY294002), ERK (PD98059), JNK (JNK-in) and p38 MAPK (SB203580) inhibitors, were employed to examine if the corresponding kinase is involved in the phosphorylation of the Ser^194^-Pro motif of Grb7. Among the inhibitors used, the JNK inhibitor exhibited an apparent interference of the interaction between Pin1 and Grb7 (Fig 3A and S1 Fig). In addition to the pharmacological inhibition of JNK activity, we found that the interaction between Pin1 and Grb7 was significantly reduced in the MAPK8 knockdown (shRNA against JNK mRNA) cells compared to the mock control cells (Fig 3B). In accordance with the phosphorylation of the Ser^194^-Pro motif-mediated Grb7 interaction with Pin1, we found that the phospho-Ser/Thr-Pro motif of Grb7 was affected by JNK (Fig 3C). On the other hand, MAP kinase kinase 7 (MKK7), which functions upstream of JNK [25], was employed to test whether increased phosphoylation of JNK facillitates the Grb7 phosphorylation on Ser/Thr–Pro motifs as well as the Pin1-binding ability of Grb7. As expected, both the Grb7 phosphorylation on Ser/Thr–Pro motifs and the Pin1-binding ability of Grb7 were increased in MKK7-transfected cells in comparison to mock transfectants (Fig 3D). Moreover, the effects of MKK7-mediated JNK activation on phosphorylated Ser/Thr–Pro motifs and Pin1-binding ability of Grb7 were markedly reduced with the Grb7 S194A mutant in comparison to wild-type Grb7 (Fig 3E). Meanwhile, we also observed that the Grb7 S194A mutant failed to interact with JNK, indicating that Grb7 binding to JNK might occur prior to Grb7 phosphorylation at the Ser^194^-Pro motif by JNK (Fig 3F). These results suggest that JNK acts as an upstream kinase in regulating the phosphorylation of Grb7 on the Ser^194^-Pro motif.

### Pin1 negatively regulates the stability of Grb7 protein

**Fig 4:**
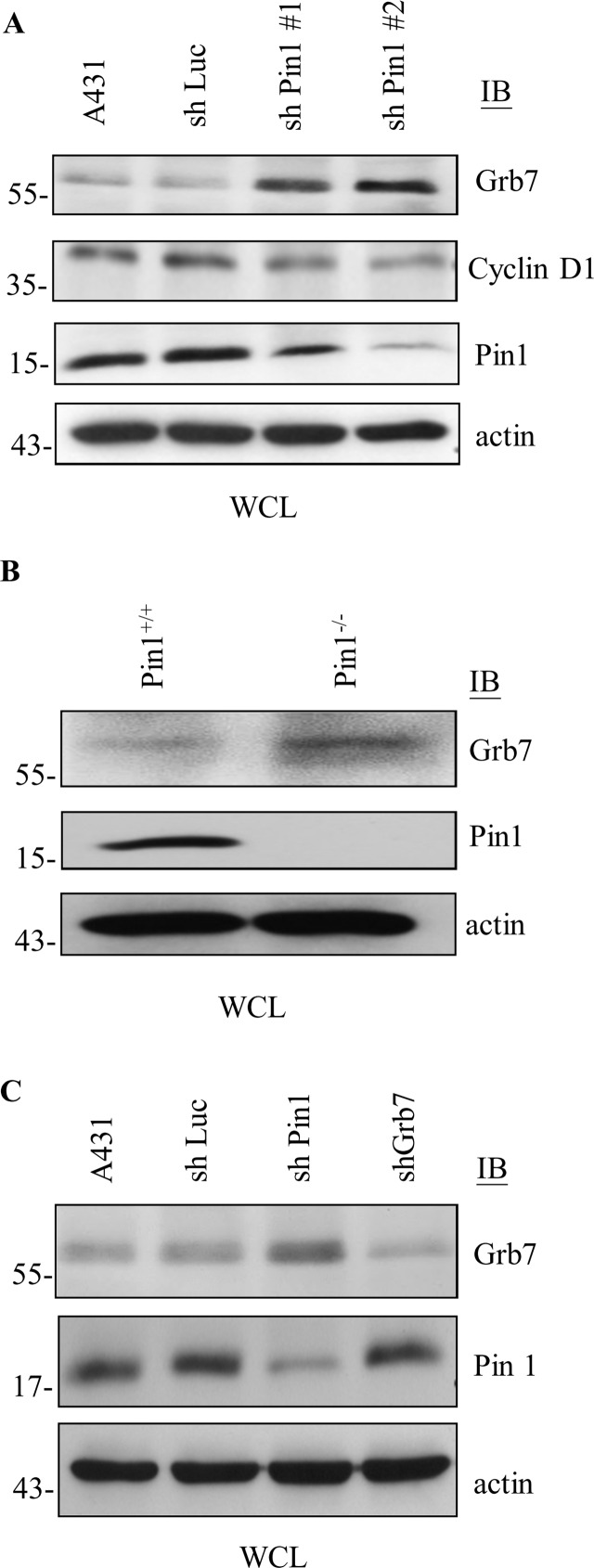
Pin1 reduces Grb7 protein stability. (A) Cell lysates from shLuc- or shPin1-infected (shPin1 #1 or shPin1 #2) A431 cells were collected and subjected to Western blotting with anti-Pin1, anti-Grb7, or anti-Cyclin D1 antibody to examine the protein expression of the indicated molecules. (B) Cell lysates from Pin1 wild-type (Pin1^+/+^) and knockout (Pin1^-/-^) mouse embryonic fibroblasts were collected and subjected to Western blotting with anti-Pin1 or anti-Grb7 antibody. (C) Cell lysates from shLuc-, shPin1-, or shGrb7-infected A431 cells were collected and subjected to Western blotting with anti-Pin1 or anti-Grb7 antibody. The results demonstrated that the protein expression of Grb7 was increased in the Pin1 knockdown condition. Each experiment was repeated at least three independent times and the representative blots were shown in A-C.

**Fig 5:**
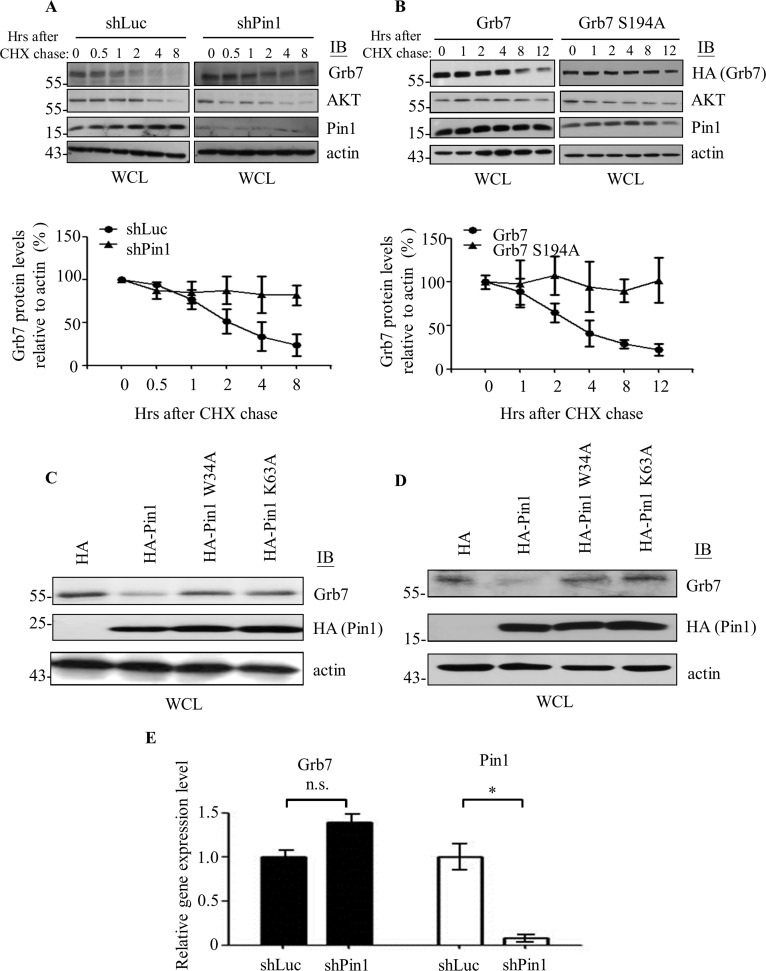
Pin1 targets Grb7 for proteasome-mediated degradation. (A) shLuc- or shPin1-infected A431 cells were treated with cycloheximide (CHX, 25 μg/ml) for the indicated times to stop protein translation. Cell lysates were collected and subjected to Western blotting with anti-Pin1, anti-Grb7, or anti-AKT antibody. shLuc-infected cells were used as a control. The protein expression of AKT, known to be modulated by Pin1, was used as a control. (B) A431 cells that co-expressed with HA-Grb7 or its S194A point mutant were treated with cycloheximide (CHX, 25 μg/ml). Cell lysates were collected and subjected to Western blotting with anti-Pin1, anti-HA for HA-Grb7, or anti-AKT antibody. Quantification of the Grb7 protein level derived from the cycloheximide pulse-chase assay is shown. Results were normalized to actin, and the Grb7 protein expression level is shown as the percent change from time zero, which was set as 100%. (C) shPin1-infected A431 cells or (D) Pin1 knockout (Pin1^-/-^) mouse embryonic fibroblasts were re-introduced with HA-Pin1 or its point mutants, W34A and K63A. Cell lysates were collected and subjected to Western blotting with anti-HA for HA-Pin1, anti-Grb7, or anti-actin antibody to detect the protein expression level of the indicated molecules. The results showed that Pin1-modulated Grb7 protein stability depends on the binding ability and peptidyl-prolyl cis-trans isomerase activity of Pin1. (E) The mRNA levels of GAPDH, Grb7, and Pin1 from shLuc- and shPin1-infected A431 cells were detected by quantitative RT-PCR. The mRNA levels of Grb7 and Pin1 were normalized to GAPDH. Student’s *t*-test was used for statistical analysis. *, *p* < 0.05.

Next, we investigated the consequence of the Pin1-Grb7 interaction. We found that the protein level of Grb7 was markedly higher in the Pin1 knockdown cells compared to the mock control cells (Fig 4A). Such outcome is consistent with Pin1 being capable of modulating the stability of its substrates like cyclin D1 (Fig 4A) [13, 18, 26]. Moreover, the protein stability of Grb7 was higher in embryonic fibroblasts derived from Pin1 knockout mouse compared to wild-type (Fig 4B). On the other hand, the protein stability of Pin1 was not influenced by Grb7 (Fig 4C), indicating that Grb7 is an authentic substrate for Pin1. In addition, we examined whether Grb7 protein stability enables modulation by Pin1 in a steady state utilizing a cycloheximide (CHX) chase assay. As expected, Grb7 protein stability decreased with a half-life of approximately 2 hours in the presence of Pin1; whereas, depletion of Pin1 prolonged the half-life of Grb7 protein to more than 8 hours (Fig 5A). Moreover, mutation at S194A of Grb7, which causes a deficiency in interaction with Pin1, resulted in reversing the instability of the wild-type Grb7 (Fig 5B). In contrast, no apparent change was observed at the expression level of endogenous Pin1 protein while Grb7 was knocked down, indicating that Pin1 acts as an upstream negative regulator of Grb7.

To further elucidate the attribute of Pin1 in modulating Grb7 protein stability, we examined Grb7 protein stability while overexpressing wild-type Pin1 or its W34A (pSer/Thr-Pro motif binding defect) or K63A (peptidyl-prolyl cis-trans isomerase defect, PPIase defect) mutantin Pin1 knockdown cells (Fig 5C) or Pin1 knockout mouse embryonic fibroblasts (Fig 5D). Reduction of the Grb7 protein level was only found in the wild-type Pin1-overexpressing cells, but not in the W34A or K63A mutant-expressing cells. This result indicated that the reduction of Grb7 protein stability indeed requires both the binding (via the WW domain of Pin1) and PPIase activities of Pin1. Notably, this negative regulatory mechanism of Grb7 protein expression by Pin1 is not modulated at the transcriptional level due to the lack of a significant difference in the Grb7 mRNA level in shPin1-infected versus shLuc-infected A431 cells using a quantitative real-time PCR analysis (Fig 5E). Together, our results suggested that both protein binding ability and isomerization by Pin1 are cricial for the negative regulation of Grb7 protein stability rather than the transcriptional expression of Grb7 mRNA. Collectively, our findings reveal that Grb7 protein stability could be modulated by the peptidyl-prolyl cis-trans isomerase activity of Pin1 via a physical interaction.

### Pin1 negatively regulates the Grb7 protein stability through ubiquitin-proteasome proteolysis

**Fig 6:**
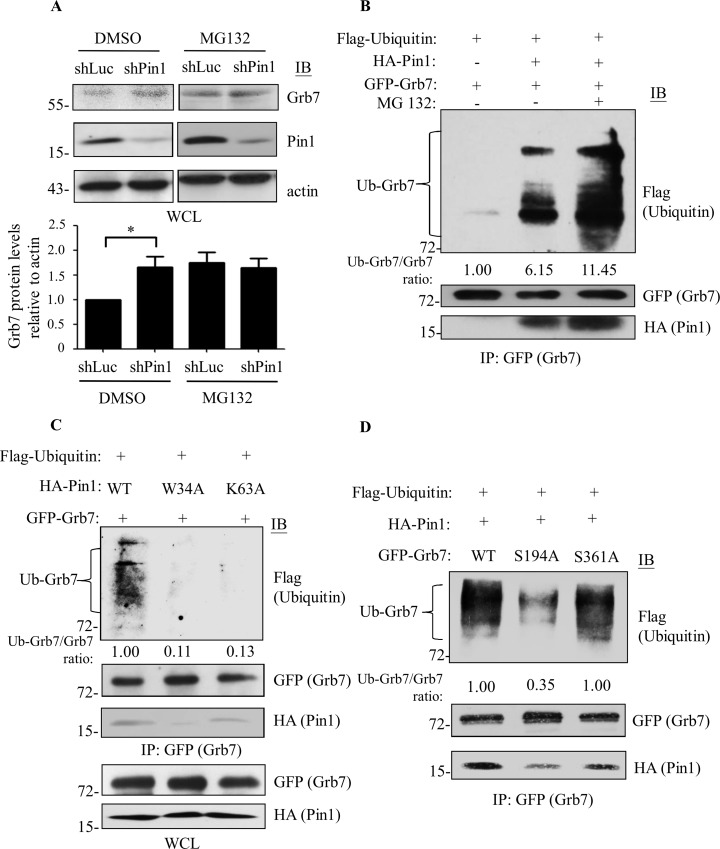
Pin1 stimulates the ubiquitination of Grb7. (A) shLuc- or shPin1-infected A431 cells were treated with MG132 (10 μM) for 8 hr to block the function of the proteasomes. Cell lysates were collected and subjected to Western blotting using anti-Pin1 and anti-Grb7 antibodies. DMSO-treated cells were used as a control. Student’s t-test was used for statistical analyses. Results were represented the mean ± SD of at least three independent experiments. *, *p* < 0.05 was considered significant differences among the experimental groups. (B) Flag-ubiquitin and GFP-Grb7 were co-transfected with or without HA-Pin1 into HEK293 cells. Twenty-four hours after transfection, cells were treated with or without MG132 (10 μM) for an additional 12 hr. Cell lysates were immunoprecipitated by anti-GFP antibody against GFP-Grb7, and co-immunoprecipitated Pin1 and ubiquitinated Grb7 were visualized by anti-HA and anti-Flag antibodies, respectively. (C) Pin1 knockout (Pin1^-/-^) mouse embryonic fibroblasts were co-transfected with Flag-ubiquitin, GFP-Grb7, and HA-Pin1 or its point mutation mutants, W34A and K63A. Twenty-four hours after transfection, cells were treated with MG132 (10 μM) for an additional 10 hr. Cell lysates were immunoprecipitated by anti-GFP antibody against GFP-Grb7, and co-immunoprecipitated Pin1 and ubiquitinated Grb7 were visualized by anti-HA and anti-Flag antibodies, respectively. (D) HEK293 cells were co-transfected with Flag-ubiquitin, HA-Pin1, and GFP-Grb7 or its Ser to Ala point mutants, S194A and S361A. Twenty-four hours after transfection, cells were treated with MG132 (10 μM) for an additional 12 hr. Cell lysates were immunoprecipitated by anti-GFP antibody against GFP-Grb7, and co-immunoprecipitated Pin1 and ubiquitinated Grb7 were visualized by anti-HA and anti-Flag antibodies, respectively. Each experiment was repeated at least three independent times and the representative blots were shown in A-D.

Furthermore, we sought to elucidate the underlying mechanism by which Pin1 negatively modulates Grb7 protein stability. Several studies have demonstrated that Pin1 enables control of protein stability via ubiquitin-mediated proteolysis, for example, NF-kB, interferon-regulatory factor 3 [19, 27]. As shown in Fig 6A, A431 cells were treated with a proteasome inhibitor, MG-132, which prolonged Grb7 protein stability similar to that of the Pin1 knockdown cells. This result indicated that Pin-modulated degradation of Grb7 protein is probably mediated by the proteasome pathway. To further test this possibility, we found that co-transfection of Pin1 with Grb7 indeed increased ubiquitinated Grb7 protein compared to Grb7 alone (Fig 6B). Not surprisingly, a significant accumulation in ubiquitinated Grb7 protein was detected upon the presence of MG132. These results suggest that Pin1 reduces the stability of Grb7 protein via an ubiquitin-dependent proteasome protein degradation cascade.

Moreover, the promotion of ubiquitinated Grb7 protein was impeded upon co-transfection with W34A (the pSer/Thr-Pro motif binding deficient mutant) or K63A (the PPIase deficient mutant) mutant of Pin1 compared to the wild-type Pin1 (Fig 6C). This result proposed essential roles for both the binding ability and PPIase activity of Pin1 in the regulation of Grb7 ubiquitination and subsequent proteasome degradation. Similarly, the less ubiquitination of Grb7 S194A mutant was due to incapable of interacting with Pin1 compared with wild-type Grb7 or its S361A mutant (Fig 6D). Taken together, our data reveal a novel Grb7 regulatory mechanism through the Pin1-mediated ubiquitin/proteasome cascade.

### Effects of the Pin1/Grb7 complex on cell cycle progression

**Fig 7:**
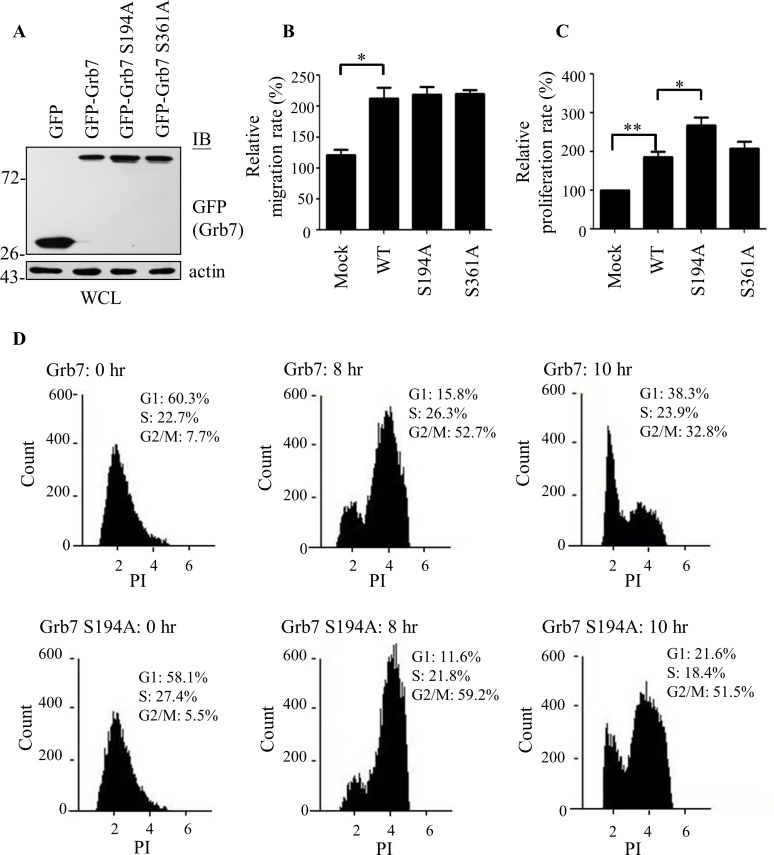
Effects of the Pin1/Grb7 complex on cell functions. (A) Cell lysates from A431 cells expressing GFP-Grb7 or its Ser to Ala point mutation mutants, S194A and S361A, were collected and subjected to Western blotting using anti-GFP antibody against GFP-Grb7. A431 cells expressing GFP-Grb7 or its Ser to Ala point mutation mutants, S194A and S361A, were subjected to a modified Boyden chamber cell migration assay (B) and cell proliferation analysis by BrdU incorporation (C) as described in the Materials and Methods. GFP-expressing A431 cells were used as the mock. All results are shown as the mean ± SD of three independent experiments. *, *p* < 0.05, **, *p* < 0.01. (D) A431 cells expressing GFP-Grb7 or Grb7-S194A were collected at the indicated time points post-release from thymidine synchronization. The cell cycle progression of GFP-positive cells was measured after propidium iodide staining by fluorescence-activated cell sorting analysis.

Given the essential role of Grb7 in cell cycle regulation and cell migration, we then explored the effect of Pin1 interaction with Grb7 on these cellular functions. First, the A431 cells stably expressing wild-type, S194A or S361A mutant Grb7 (Fig 7A) were subjected to a cell migration assay toward fibronectin (10 μg/ml) or EGF (20 ng/ml, unpublished data) in a modified Boyden chamber. Although Grb7 overexpression intrinsically enhanced cell migration compared to the mock control, there was no increase in cell migration by overexpression of the S194A mutant of Grb7 compared to the wild-type Grb7 (Fig 7B). This result indicated that the consequence of Pin1 interaction with Grb7 is not essential for cell migration. On the other hand, the S194A mutant of Grb7 promoted cell proliferation much more than the wild-type or S361A mutant of Grb7, suggesting the involvement of Pin1 in modulating Grb7-mediated cell cycle progression (Fig 7C). To further investigate the role of Grb7 in cell progression, the influence of wild-type Grb7 and the S194A mutant of Grb7 on the cell cycle progression was compared using fluorescence-activated cell sorting analyses. Despite a moderate increase in entering the G2/M transition at 8 hr after the release of thymidine block, the S194A mutant of Grb7 induced a remarkable delay in exiting M phase in comparison with wild-type Grb7 at 10 hr after the release of the thymidine block (Fig 7D), suggesting the involvement of the Pin1-mediated Grb7 stability in controlling the M phase progression. Together, our data provide new insight into the regulatory network of Grb7-mediated cell cycle progression regulated by Pin1.

## Discussion

Grb7 is a multi-domain adaptor protein positively modulated by growth factor or integrin signaling; whereas, negative regulation of Grb7-mediated signaling remains elusive. Here, we illustrated an intrinsic and negative regulatory mechanism of Grb7 by Pin1. Pin1 facilitates the post-phosphorylation conformational change of protein substrates by isomerizing phospho-Ser/Thr-Pro motifs to modulate protein stability, thereby playing an essential role in the regulation of diverse cellular functions, including enzymatic activities, protein-protein interaction, localization, and protein stability [13]. Consistently, we found that the phospho-Ser^194^-Pro motif of Grb7 enables the physical interaction with Pin1 (Fig 2C) in a JNK-dependent manner. Subsequently, the peptidyl-prolyl *cis/trans* isomerase activity of Pin1 results in Grb7 protein instability via a ubiquitin/proteasome-dependent proteolysis cascade (Fig 6A and 6B). As a result, Grb7-mediated cell proliferation, the exit from M phase in particular, is modulated by Pin1. Our results, for the first time, highlight a novel regulation of Grb7 by Pin1 negatively modulating Grb7 protein stability and Grb7-mediated cell cycle progression.

We found that JNK, but not p38, ERK, CDK1 or GSK3, enables binding to the Ser-Pro motif and further inducing phosphorylation at the Ser^194^ residue of Grb7 protein (Fig 3C, 3E and 3F). Consistent with the above results, Grb7 amino acid sequence (192-KSSPHS-197) was found to contain minimal phosphoaccepter Ser-Pro (SP) motif for phosphorylation by JNK [28, 29]. Our results also indicated that MKK7-mediated JNK phosphorylation involved in the regulation of the Grb7 phosphorylation on Ser/Thr–Pro motifs and the Pin1-binding ability of Grb7 (Fig 3D and 3E), is a novel regulatory mechanism of Grb7 by MAPK siganling. Moreover, the phosphorylation on Ser or Thr residues of substrates by MAPKs usually facilitates their binding to E3 ubiquitin ligases, leading to phosphorylation-dependent ubiquitin-proteasome proteolysis that can accelerate protein turnover [30]. In line of the above, we conclude that JNK-dependent post-phosphorylation modification of Grb7 by Pin1 may promote the turnover of Grb7 protein in an ubiquitin-proteasome proteolysis-mediated process (Figs 3C, 3E, 6B and 6D). In accordance with our observation, phosphorylation of the serine in proteins, e.g., insulin receptor substrate or steroid/thyroid/retinoid receptor superfamily TR3, by JNK indeed facilitates the isomerization with these proteins by Pin1 and, subsequently enhances protein turnover via ubiquitin-proteasome proteolysis [30–32].

Although the positive regulation of Grb7 is well established, the negative regulatory mechanism for Grb7 is elusive. Protein degradation via the ubiquitin-proteasome proteolysis pathway is emerging as an ubiquitous and underlying mechanism in regulating intracellular signaling [33]. In the present study, we proposed that the intrinsic conformational switch of Grb7 that occurred on the phospho-Ser^194^-Pro motif by Pin1 might facilitate the accessibility to E3 ubiquitin ligase, thereby contributing to the ubiquitination of Grb7 (Fig 6B, 6C and 6D). As expected, the point mutation on the Ser^194^ residue (S194A) of Grb7 abolished Pin1-binding ability and isomerization of Grb7 by Pin1 (Fig 2C). Similarly, the WW domain mutation and the PPIase-deficient mutation of Pin1 resulted in the stabilization and accumulation of Grb7 protein even in the steady-state (Fig 5B). Nonetheless, the E3 ubiquitin ligase for Grb7 remains unclear; the NEDD4 family of E3 ubiquitin ligases, i.e., neural precursor cell expressed developmentally down-regulated protein 4 (NEDD4) or its close relative NEDD4-L, are reported to associate with the Grb7-related family member, Grb10. Therefore, it is interesting to test if any member of the NEDD4 family E3 ubiquitin ligases participates in Grb7 ubiquitination. Moreover, we also found that ubiquitin specific peptidase 8 (USP8), as a deubiquitinating enzyme [34], directly interacted with Grb7 in our yeast two-hybrid screening (unpublished data), suggesting a complicated regulation between ubiquitination and deubiquitination in response to the intrinsic conformational change of Grb7 by Pin1. In fact, the antagonized functionality between the NEDD4 family and USP8 has been proposed in a coordinate fashion to be involved in protein ubiquitination and deubiquitination [35]. Further studies are needed to explore how the NEDD4 family of E3 ubiquitin ligases and the USP8 deubiquitination peptidase regulate Grb7 protein stability.

Recent studies have suggested, a critical role of Grb7 in cell proliferation [2, 8, 36]. For instance, FAK-mediated phosphorylation of specific tyrosine residues of Grb7 promotes cell proliferation via activation of the MAPK cascade [8]. In contrast, knockdown of Grb7 by lentiviruses encoding Grb7 small-hairpin RNAs (shRNA) reduces the cell proliferation as well as impairs cell growth [4, 8]. Here, we illustrated that Pin1 is involved in Grb7-mediated cell cycle progression (Fig 7C and 7D), which is consistent with previously reported function of Pin1 as an important mediator of cell cycle regulators, such as cyclin D1, p53, and topoisomerase [12, 17, 20, 21]. In spite of Pin1-mediated cyclin D1 stability which is required for cell cycle progression [37], depletion of Pin1 induces mitotic arrest; whereas, overexpression of Pin1 leads to an improper transition of the cell cycle from G2 phase to M phase [38, 39]. In accordance with previous studies [17, 38, 39], our study also suggested that Pin1-modulated Grb7 protein stability involves in the G2/M phase transition (Fig 7D). Indeed, Pin1 and the upstream regulator of the Pin1/Grb7 complex, JNK, have been reported to affect cyclin/CDKs in regulating the G2/M phase transition [17, 40, 41]. Although detailed regulatory mechanisms of Grb7 on the G2/M phase transition need to be further investigated, previous studies [17, 40, 41] and ours suggested that JNK-mediated Pin1/Grb7 complex could affect cyclin/CDKs in regulating cell cycle. Moreover, the dysregulation of Grb7 by Pin1 gives rise to a moderate increase in cells entering the G2/M phase transition; while, the prolonged effect of accumulative Grb7 results in a delayed exit from M phase (Fig 7D). Our finding also implicated an expanding role for the coordinate involvement of Pin1 and Grb7 in controlling M phase.

In spite of the expression and hypophosphorylation of Pin1 often correlated with human oncogenesis [14, 21, 42–45], depletion of Pin1 expression has also been reported to contribute to tumorigenesis, as a result of stabilizing c-Myc or cyclin E [15, 20, 46]. In addition, Pin1 also leads to the timely accumulation of p53 protein and promotes p53-mediated cell cycle arrest in response to genotoxic stresses [12, 18]. Indeed, stress-induced phosphorylated p53 facilitates its Pin1 binding ability, followed by promoting the dissociation of p53 from Mdm2 [47] or inhibitor of apoptosis-stimulating protein (iASPP) [48], which in turn affects p53-mediated apoptotic response. Recently, Pin1 has been reported to promote the stress-induced mitochondrial accumulation and functional activation of p53, followed by facilitating the association between p53 and the Bcl-2 family of mitochondrial permeability regulators [49]. Additionally, Pin1 is also involved in the conformational change of p53-related protein, p73, resulting in its stabilization and functional activitation [50]. These studies suggested that Pin1 functions as a negative regulator of cell proliferation. The contradictory role of Pin1 in oncogenesis suggests that Pin1 could act as a tumor promoter or conditional tumor suppressor depending on the Pin1 expression level, cell types and the signal transduction process that is regulated [14]. As a result of the Pin1-mediated Grb7 degradation (Fig 4), ablation of Pin1/Grb7-coordinated cell cycle progression may lead to abnormality of cell proliferation in relation to certain pathogeneses in humans. Although the clinical relevance of Pin1 and Grb7 expression during oncogenesis remains to be examined, both molecules have been known to be often involved in cancer progression [2, 4, 8, 13, 46, 51–53].

In summary, this study reveals that a physical interaction between Grb7 and Pin1 is involved in cell cycle progression via regulating Grb7 protein stability. We also illustrated an underlying mechanism in whch JNK MAPK is responsible for the phosphorylation of Grb7 on the Ser^194^-Pro motif and the post-phosphorylation modification of Grb7 by the peptidyl-prolyl *cis/trans* isomerase activity of Pin1, which then confers to the Grb7 ubiquitin-proteasome proteolysis. Our findings provide a novel mechanism for the regulation of Grb7 by Pin1 in cell cycle progression.

## Supporting Information

S1 Fig The effects of pharmacological agents on indicated signal molecules. A431 cells were treated with PD98059 (20 μM), JNK-in (25 μM), SB203580 (10 μM), LY294002 (10 nM), CGP74514A (5 μM), TDZD-8 (50 μM), or SB-415286 (20 μM) for 1 hr. Cell lysates were collected and subjected to Western blot analysis to analyze the phosphorylation and expression of indicated signal molecules.
(PDF)
