# Supplementary figures and images for: Grb7 Protein Stability Modulated by Pin1 in Association with Cell Cycle Progression
Source: PLoS One. 2016 Sep 22;11(9):e0163617. doi: 10.1371/journal.pone.0163617 (PMC5033455; doi:10.1371/journal.pone.0163617)

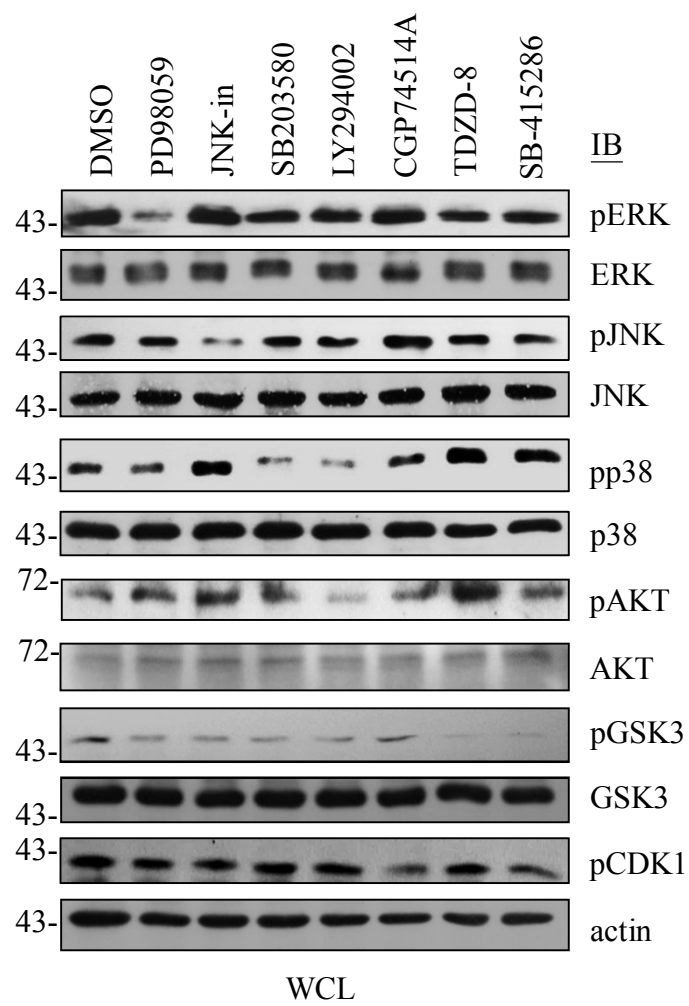

Supplement: S1 Fig — A431 cells were treated with PD98059 (20 μM), JNK-in (25 μM), SB203580 (10 μM), LY294002 (10 nM), CGP74514A (5 μM), TDZD-8 (50 μM), or SB-415286 (20 μM) for 1 hr. Cell lysates were collected and subjected to Western blot analysis to analyze the phosphorylation and expression of indicated signal molecules. (PDF) [file pone.0163617.s001.pdf]
